# Visceral Leishmaniasis and HIV Coinfection in Latin America

**DOI:** 10.1371/journal.pntd.0003136

**Published:** 2014-09-18

**Authors:** José Angelo Lindoso, Gláucia Fernandes Cota, Alda Maria da Cruz, Hiro Goto, Ana Nilce Silveira Maia-Elkhoury, Gustavo Adolfo Sierra Romero, Márcia Leite de Sousa-Gomes, Joanna Reis Santos-Oliveira, Ana Rabello

**Affiliations:** 1 Instituto de Infectologia Emilio Ribas, São Paulo, São Paulo, Brasil; 2 Laboratório de Soroepidemiologia (LIM-38) Hospital das Clínicas da Faculdade de Mediciina da Universidade de São Paulo, São Paulo, São Paulo, Brazil; 3 Instituto de Medicina Tropical de São Paulo, Universidade de São Paulo, São Paulo, São Paulo, Brazil; 4 Hospital Eduardo de Menezes, Fundação Hospitalar do Estado de Minas Gerais (FHEMIG), Belo Horizonte, Minas Gerais, Brazil; 5 Centro de Pesquisa René Rachou, Fundação Oswaldo Cruz (FIOCRUZ), Belo Horizonte, Minas Gerais, Brazil; 6 Laboratório Interdisciplinar de Pesquisas Medicas, Instituto Oswaldo Cruz–FIOCRUZ, Rio de Janeiro, Rio de Janeiro, Brazil; 7 Disciplina de Parasitologia/FCM-UERJ, Manguinhos, Rio de Janeiro, Rio de Janeiro, Brazil; 8 Departamento de Medicina Preventiva da Faculdade de Medicina, Universidade de São Paulo, São Paulo, São Paulo, Brazil; 9 Pan American Health Organization-World Health Organization (PAHO-WHO), Duque de Caxias, Rio de Janeiro, Brazil; 10 Núcleo de Medicina Tropical, Universidade de Brasilia, Distrito Federal, Brazil; 11 Instituto Nacional de Ciência e Tecnologia de Avaliação de Tecnologia em Saúde, Porto Alegre, Rio Grande do Sul, Brazil; 12 Fundação de Amparo à Pesquisa do Estado do Amazonas (FAPEAM), Manaus, Amazonas, Brazil; 13 Ministério da Saúde do Brasil, Brasília, Distrito Federal, Brazil,; National Institute of Allergy and Infectious Diseases, United States of America

## Abstract

Visceral leishmaniasis (VL) is an endemic zoonotic disease in Latin America caused by *Leishmania (Leishmania) infantum*, which is transmitted by sand flies from the genus *Lutzomyia*. VL occurs in 12 countries of Latin America, with 96% of cases reported in Brazil. Recently, an increase in VL, primarily affecting children and young adults, has been observed in urban areas of Latin America. The area in which this spread of VL is occurring overlaps regions with individuals living with HIV, the number of whom is estimated to be 1.4 million people by the World Health Organization. This overlap is suggested to be a leading cause of the increased number of reported VL-HIV coinfections. The clinical progression of HIV and *L. infantum* infections are both highly dependent on the specific immune response of an individual. Furthermore, the impact on the immune system caused by either pathogen and by VL-HIV coinfection can contribute to an accelerated progression of the diseases. Clinical presentation of VL in HIV positive patients is similar to patients without HIV, with symptoms characterized by fever, splenomegaly, and hepatomegaly, but diarrhea appears to be more common in coinfected patients. In addition, VL relapses are higher in coinfected patients, affecting 10% to 56.5% of cases and with a lethality ranging from 8.7% to 23.5% in Latin America, depending on the study. With regards to the diagnosis of VL, parasitological tests of bone marrow aspirates have proven to be the most sensitive test in HIV-infected patients. Serologic tests have demonstrated a variable sensitivity according to the method and antigens used, with the standard tests used for diagnosing VL in Latin America displaying lower sensitivity. For this review, few articles were identified that related to VL-HIV coinfections and originated from Latin America, highlighting the need for improving research within the regions most greatly affected. We strongly support the formation of a Latin American network for coinfections of *Leishmania* and HIV to improve the consistency of research on the current situation of VL-HIV coinfections. Such a network would improve the collection of vital data and samples for better understanding of the clinical manifestations and immunopathogenic aspects of VL in immunosuppressed patients. Ultimately, a concerted effort would improve trials for new diagnostic methodologies and therapeutics, which could accelerate the implementation of more specific and effective diagnosis as well as public policies for treatments to reduce the impact of VL-HIV coinfections on the Latin American population.

## Introduction

To carry out this narrative review, a systematic search in major databases concerning the Americas (Medline and Literatura Latino-Americana e do Caribe em Ciências da Saúde [LILACS]) was performed specifically for published reports on *Leishmania*-HIV coinfection. Additionally, documents regarding leishmaniasis produced by the Pan American Health Organization/World Health Organization (PAHO/WHO) were obtained (Table 1). There was a dearth of reports, and most of the available publications were produced in Brazil, which currently has the highest incidence and disease burden of both infections in the Americas. This fact limits the application of the present review to the Brazilian scenario. Nonetheless, epidemiological and sociodemographic features, as well as diagnosis and clinical and therapeutic approaches, have similarities with other Latin American countries.

**Table 1:** Studies reporting visceral leishmaniasis-HIV coinfection in the Americas, 1990–2013.

Publication Year	Author	Country	Year-Period	Study Design
1990	Nicodemo et al. [8]	Brazil	1989	Case report
1995	Hernandez et al. [59]	Venezuela	1992	Case report
2000	Ramos-Santos et al. [67]	Mexico	1997	Case report
2001	Chehter et al. [58]	Brazil	1995	Case report
2002	Bittencourt et al. [64]	Brazil	2000	Case report
2002	Orsini et al. [66]	Brazil	1997	Case report
2003	Rabello et al. [10]	Brazil	2003	Review
2008	Roselino et al. [52]	Brazil	1995	Case report
2008	Maia-Elkhoury et al. [12]	Brazil	2001–2005	Case series
2009	Carnaúba Jr et al. [65]	Brazil	2004	Case report
2009	Daher et al. [74]	Brazil	2003–2007	Case series
2010	Alexandrino-de-Oliveira et al. [41]	Brazil	2000–2006	Case series
2010	Santos-Oliveira et al. [46]	Brazil	Unknown	Case series
2011	Nascimento et al. [14]	Brazil	1990–2008	Case series
2011	Sousa-Gomes et al. [15]	Brazil	2007–2008	Case series
2011	Santos-Oliveira et al. [47]	Brazil	2006	Case series
2012	Souza et al. [73]	Brazil	2000–2005	Case series

## Epidemiology

Visceral leishmaniasis (VL) is a zoonotic infection common in the Americas that is caused by *Leishmania (Leishmania) infantum*, a parasite transmitted through the bite of female hematophagous sand flies belonging to the genus *Lutzomyia* [1]. Between 2001 and 2011, 38,808 VL cases were recorded in the Americas. Although these cases are distributed in 12 countries, including recent reports from Paraguay and Argentina, 96% of American VL cases occur in Brazil (37,503 cases). In 2011, when eight countries reported 4,004 VL cases in Latin America, the VL incidence was 2.02 cases per 100,000 inhabitants. The affected population is primarily men (61.7%), and 36% of all patients are children younger than 5 years [2]. Due to expected underreporting of 11.1% to 41.2%, a more realistic estimate varies from 4,500 to 6,800 cases per year [3].

Acquired immunodeficiency syndrome (AIDS) is considered an emerging disease in this region because of its geographical distribution and the extent of damage to the population. The World Health Organization (WHO) estimated that in 2011 nearly 1.4 million people were living with HIV in Latin America. In this region, the number of new HIV infections remained steady (12%), with approximately 83,000 new cases in 2011 compared to 93,000 in 2001, in contrast to the 28% reduction in the occurrence of new cases worldwide [4], [5].

The spreading of HIV infection to rural areas and the urbanization of the *L. infantum* transmission cycle could have influenced the epidemiology and natural history of these diseases, including acceleration of the clinical progression of both due to cumulative immunosuppression. Additionally, the presence of HIV infection in endemic areas of VL increases the risk of development of this disease [6], [7].

The first American *Leishmania*-HIV-coinfected patient was reported in Brazil [8] 5 years after the first world report in 1985 in southern Europe [9]. Since then, many other coinfected cases have been reported in other Latin American countries (Table 1). More recently, Argentina and Paraguay, where VL is expanding, have also reported VL cases [2].

**Figure 1:**
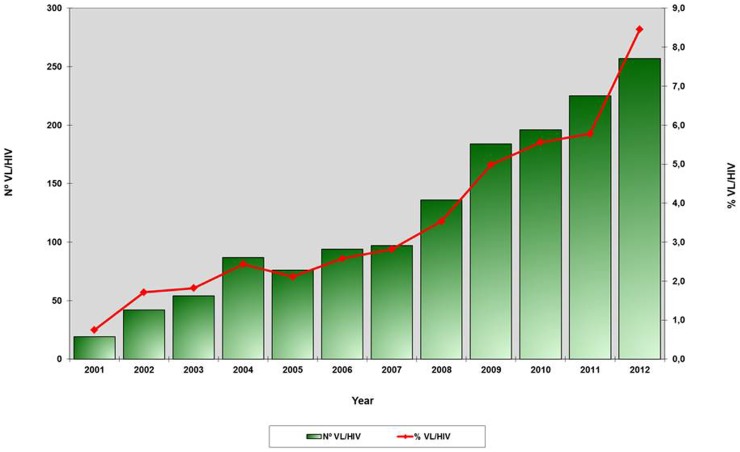
Absolute number and relative proportion of coinfected *Leishmania*-VL cases among the total number of VL cases reported. Cases and proportion of visceral leishmaniasis-HIV coinfection in Brazil from 2001 to 2012. Source: Surveillance Secretary of Health/Ministery of Health in Brazil.

In Brazil, a first appraisal of *Leishmania*-HIV coinfection was performed in 2003. This study gathered 91 *Leishmania*-HIV-coinfected patients, 37% (33/91) of whom presented with VL [10]. Just after this first initiative, through a global network, WHO developed a standardized data collection form and encouraged a surveillance and reporting system of VL-HIV coinfection in different continents. At that time, the Brazilian Ministry of Health strengthened the national HIV and leishmaniasis reference centers to improve the early diagnosis and appropriate treatment of *Leishmania*-HIV coinfection, including the issuing of specific guidelines. Additionally, the surveillance system was reorganized. Information on coinfection was included in the individual HIV and leishmaniasis obligatory patient report forms, and the two national databases (AIDS and leishmaniasis) were aligned to permit cross analysis. Thereafter, available data have shown an increasing trend in the VL-HIV coinfection incidence in Brazil. In 2001, approximately 0.7% of all VL cases were reported in HIV-infected patients, while in 2012 this percentage increased to 8.5% (Figure 1) [11]. Using cross database linkages of the two national reporting systems between 2001 and 2005, a second Brazilian VL-HIV coinfection survey was carried out and identified 176 VL-AIDS patients (1.1% of all VL cases reported in Brazil), 78% (137/176) being adult males with a median age of 38 years [12].

In contrast to what is observed in Europe, where HIV transmission through intravenous injection in drug users represents the major route of transmission [6], in the Americas the sexual route is the most frequent HIV-transmission category among VL-HIV coinfected patients [12]–[14].

With respect to the geographical distribution of VL-AIDS, Brazilian studies based on national data reinforce the conclusion that the increasing numbers of VL-HIV coinfected cases is favored by the overlap of regions that harbor these infections. Between 2001 and 2005, 53% of VL-HIV-coinfected patients were reported in northeastern Brazil, a region that accounted for 73% and 56% of the total number of VL cases at the beginning and the end of that period, respectively, followed by the Southeast and Midwest with 29.1% and 10.8% of cases, respectively [12]. According to [15], using the national reporting system database, 38.1% of all VL-HIV-coinfected Brazilian patients between 2007 and 2008 were reported in the Northeast, 38.1% in the Southeast, and 16.2% in the Midwest, following the expansion of VL toward these regions.

Despite the increasing incidence in recent years, it is recognized that the actual magnitude of VL-HIV coinfection in the Americas is ignored. In recent years, the Brazilian HIV-AIDS Program made a remarkable effort to increase the coverage of HIV testing. This policy includes offering an HIV test as part of the routine evaluation of patients who suffer conditions that might be associated with HIV-associated immunodeficiency, such as VL. This effort improves the detection of VL-HIV coinfection cases and may partially explain the increasing trend currently observed in Brazil [16].

However, even with HIV testing becoming increasingly available, achieving adequate coverage of the exposed population remains a challenge. Furthermore, VL underreporting is a major problem in many countries in the Americas [3], [17].

## Immunopathogenesis

It is important to remember that an asymptomatic *Leishmania* infection signifies a risk for the full development of the symptomatic disease among patients infected with HIV and should prompt a close medical follow-up. The frequency of *Leishmania* infection varies among populations and diagnostic methods used, but it seems to be sizeable. Carranza-Tamayo et al. [18] found a cumulative prevalence (at least one test positive) of *Leishmania* infection of 16% among 163 HIV-infected individuals from Brasilia, Brazil. Blood samples were evaluated by means of an indirect fluorescent antibody test (IFAT), an enzyme-linked immunosorbent assay (ELISA) using a soluble antigen of *L. (L.) chagasi*, an ELISA with the rK39 antigen (ELISA-rK39), and a polymerase chain reaction (PCR) targeted to the kinetoplast DNA (kDNA) region and to the internal transcribed spacer of the ribosomal DNA (rDNA) gene. The proportion of positive results was 2.4% for the IFAT, 12.3% for the ELISA, and 4.9% for the rK39 tests. PCR in the blood was positive in three patients (1.8%). In another study [19], which used different noninvasive laboratory tests and considered those patients who presented at least one positive test to be infected, the estimated prevalence of asymptomatic infection was 20.2%. In 381 enrolled asymptomatic patients and considering only PCR, the prevalence fell to 6.3%, similar to the prevalence found by IFAT (3.9%) and by ELISA-*L. infantum* (10.8%) and in contrast to ELISA-rK39, which showed the lowest value (0.8%). In addition, 26 patients, 50% of whom had at least one positive test, were reevaluated around 16 months (9 to 20 months) after the first evaluation. All of the patients were on highly active antiretroviral therapy (HAART), and they had no symptoms and complaints related to VL. It is important to note that the mean TCD4^+^ lymphocyte count was 470 cells/L, and viral load was not detectable in 18 (69.2%) patients.

The clinical evolution of HIV and *L. infantum* infection are both profoundly dependent on the specific immune response and on the derangements of the immune system caused by both pathogens. VL-HIV coinfection can accelerate the progression of both diseases.

HIV/AIDS and VL share similar immunopathological mechanisms, including compromising the same immune compartments, such as dendritic cells (DC) and macrophages, contributing to the acceleration of the progression of both diseases. *Leishmania* infection can enhance HIV viral transcription [20]–[23].

HIV coinfection also augments *Leishmania* replication [24], [25], enhancing parasite uptake by macrophages productively infected with HIV or even uninfected bystander cells through the induction of the low-density lipoprotein -receptor-related protein (CD91/LRP-1) receptor by the parasite surface phosphatidylserine [26]. Those features can explain the elevated parasite load observed in bone marrow samples [27], [28] and peripheral blood [28], [29].

The impairment of the *Leishmania*–T lymphocyte proliferation and interferon gamma (IFN-γ) production in HIV-infected patients [30], [31] also favors the spread of the parasite. Consequently, amastigotes have been recovered from outside of the mononuclear phagocyte system [6], [32] and in the gut-associated lymphoid tissues (GALT) as well as in other nonlymphoid organs [33], [34].

The depletion of hematopoietic cells, especially T lymphocytes, in addition to intense cell immune activation is observed in both HIV/AIDS [35], [36] and VL [37]–[40]. This contributes to the reduction of CD4^+^ T lymphocytes, increased transcription of the integrated virus and an enhancement of viral load, and T cell proliferation with a consequent activated T cell death, which all together occurs in a continuous vicious circle. This activated status is maintained in VL/HIV-coinfected patients who do not recover CD4^+^ T lymphocyte counts even after specific treatment for *Leishmania*, despite antiretroviral therapy (ART) [41]. The deficient reconstitution of the T cells can lead to increased leishmaniasis relapse rates due to the inability to control parasite replication [41], [42] or even to unusual parasite localization.

Moreover, a proinflammatory cytokine storm may occur in VL in which IFN-γ, tumor necrosis factor (TNF), interleukin (IL)-1β, IL-6, IL-8, IL-17, macrophage inflammatory protein (MIP)-1β, and macrophage migration inhibitory factor (MIF) are released [29], [43]–[45]. Consistently, increased percentages of molecules associated with T cell activation, such as CD38 on CD8^+^ T cells and HLA-DR, are observed in VL/HIV patients compared to HIV/AIDS cases alone, and these molecules do not reach normal values despite undetectable viral load and clinical remission of leishmaniasis [46]. These molecules, especially those related to the risk of reactivation [47], also increase during VL relapse, suggesting that such molecules could be predictive of clinical outcome.

Together, these features raise the possibility that mechanisms other than the viral particle or *Leishmania* infection may account for this intense cellular activation, such as microbial products of luminal origin [46].

When the HIV virus spreads through mucosal dendritic cells (DCs), it enhances the early depletion of CD4^+^ T cells in the GALT, affecting mucosal immune system integrity [48]. A similar phenomenon was described in VL, most likely as a consequence of intestinal infection by *Leishmania* [29], [49]–[52]. The consequent translocation of bacterial components, such as lipopolysaccharides (LPS), may contribute to an enhancement of cellular activation by stimulating the innate and adaptive immune systems [53], creating an inflammatory environment that results in the increased proliferation and activation of T cells and causing a loss in systemic immune function [54], [55]. Then, the involvement of microbial products in addition to the *Leishmania* parasite itself could account for the immunopathogenesis of VL/HIV patients. As a consequence, secondary chemoprophylaxis for leishmaniasis or even the use of anti-inflammatory drugs or strategies to reestablish the normal gut flora can be considered to improve the prognosis of VL/HIV [29], [56].

Dysregulated cellular activation along with a proinflammatory cytokine response can lead to the death of T cell clones. Additionally, the immune senescence process generates a pool of remaining cells with poor functional quality, which then contributes to the inability of lymphocytes to proliferate and to produce the IFN-γ response to specific antigen stimuli (Santos-Oliveira, unpublished results). Clinical follow-up of coinfected patients from the active phase of the disease for long periods after treatment is essential to investigate how the major consequences of the intense cellular activation, such as immunosenescence and exhaustion of immune resources, can affect the effector immune response in *Leishmania*-HIV coinfection (Santos-Oliveira and Cota, unpublished results). In addition, as observed in other infectious diseases [57], it is expected that VL/HIV patients also experience a greater loss of thymic function. Thus, detecting and quantifying the levels of T-rearrangement excision circles in peripheral blood cells of coinfected patients (Santos-Oliveira et al., ongoing study) may help us to understand whether the inefficient immune reconstitution in VL/HIV patients can be influenced by a reduction in the ability of the thymus to reintroduce these cells into circulation.

## Clinical Aspects

As observed for other infectious agents when coinfected with HIV, *Leishmania* infection in HIV-AIDS patients may lead to a special VL clinical picture that is different from that observed in patients without HIV infection. In fact, atypical clinical cases have been reported in Latin America, including the involvement of the gastrointestinal tract (duodenum and pancreas) and kidney [52], [58], [59]. However, based on the 356 cases reported so far (Table 1), the major clinical presentation of VL-HIV-coinfected patients in the Americas is quite similar to that observed for VL in non-HIV-infected patients and in Mediterranean coinfected patients. VL and HIV were diagnosed at the same time in almost half of VL cases in this survey (66/138, 47%), suggesting that *Leishmania* spp. may have been the first opportunistic infection, prompting the HIV diagnosis. Fever occurred in 91% (317/347), and splenomegaly was present in 76% of the American patients (268/353).

The classical triad of fever, anemia, and splenomegaly was observed in a little more than half of the patients [13]. Hepatomegaly was observed in 60% of American VL-HIV published cases (164/270), which could be explained by the decrease in Kupffer cell hyperplasia due to HIV infection [60], [61]. In addition, increased fibrogenesis due to hepatic stellate cell–HIV infection can also affect liver size [62]. The results of an ongoing cohort [63] also show that *Leishmania*-HIV-coinfected patients are more frequently malnourished (60.9% versus 25%) and present a lower frequency of fever (88.9% versus 60.9%) and hepatosplenomegaly (67.4% versus 90.9%) compared with non-HIV-infected patients.

It is important to note that almost half (49%) of the reported patients had diarrhea, an unusual finding in patients not infected with HIV and suffering from VL [63]. Diarrhea may be related to infection by *Leishmania* or to HIV or coincidental intestinal opportunistic infections. Likewise, the concomitant use of antimicrobial and/or antiretroviral therapy may explain the higher frequency of intestinal disturbances. It is noteworthy that based on the largest published series from the Americas [13], some hemorrhagic phenomena, such as epistaxis, ecchymosis, and hematuria, were observed in almost a third of the patients. In the same study, the enlargement of lymph nodes was observed in 20% of coinfected patients, which is also a noteworthy aspect because it is an unusual sign in immunocompetent New World patients with VL, although it is common in East Africa.

In Latin America, cutaneous lesions caused by *L. infantum* have been rarely reported, even in immunosuppressed HIV-infected patients, in whom a higher frequency of cutaneous dissemination or post-kala-azar dermal leishmaniasis (PKDL)-like lesions, which are related to the immune reconstitution syndrome, would be expected. From the 367 VL-HIV coinfection cases gathered here, four (1.0%) patients had cutaneous involvement by a viscerotropic species of *Leishmania*, concomitant to or after the diagnosis of VL. Two patients presented cutaneous lesions, characterized as PKDL-like, after a successful treatment for VL. One patient presented confluent, miliary papules on the face, thorax, and limbs, and the other presented non-pruritic and erythematous maculopapular lesions on the face and thorax [64], [65]. In two other patients [46], [66], the cutaneous lesion was concomitant to visceral involvement (erythematous papules and plaques). In one of these patients, the strain of *Leishmania* involved in the cutaneous lesions was *L. donovani*, which was confirmed by DNA sequence analysis [46]. Surprisingly, two *Leishmania* strains were isolated from this patient: a typical *L. infantum* sequence MON-1, type A, in the bone marrow and a *L. donovani* MON-2 sequence in skin; this second strain had not been previously identified in Brazil.

Dermotropic *Leishmania* species, such as *L. braziliensis*, have been associated with the development of PKDL-like lesions in an HIV-infected patient from Venezuela, resulting in maculopapular eruption [59]. In addition, there may be a visceral manifestation of the dermotropic species *L. braziliensis* and *L. mexicana* [59], [67]. Of note, there is no record of immune reconstitution inflammatory syndrome (IRIS) in American patients infected with viscerotropic *Leishmania* species. However, the first report of IRIS in a *Leishmania* infection was from two cases of tegumentary leishmaniasis in Brazil [68].

## Laboratorial Diagnosis

Just as with clinical presentation, there is very little information about the performance of the various techniques for VL diagnosis in the Americas in HIV-coinfected patients. Despite the clearly recognized low sensitivity of serological methods among HIV-infected patients [69], there is some doubt whether one serological technique is superior to others for VL diagnosis among HIV-infected patients and whether there is a difference in test performance in different global regions. The IFAT remains the routine serological test used by public health services in Brazil. Recently, a rapid test based on the detection of antibodies against antigen rk39 was introduced in Brazil, Argentina, and Paraguay.

In the most extensive diagnostic study on *Leishmania*-HIV coinfection conducted in the Americas [70], 113 HIV-infected symptomatic patients were evaluated by an adjudication committee after clinical follow-up to establish the presence or absence of VL. The index tests were as follows: recombinant K39 antigen-based immunochromatographic test (Kalazar Detect), IFAT, direct agglutination test (DAT), and real-time polymerase chain reaction (quantitative PCR [qPCR]) based on the small subunit ribosomal of RNA (SSUrRNA) and performed in peripheral blood. Compared to a parasitological test and to the adjudication committee diagnosis or through latent class model analyses, IFAT and the rk39 dipstick test presented the lowest sensitivity (less than 60%). DAT exhibited good overall performance, and there was no significant difference between DAT and qPCR diagnosis with respect to accuracy, with both above 85%. DAT was highly sensitive and was found to be a suitable alternative to screening visceral leishmaniasis in HIV-infected patients. The ability to make most VL diagnoses without the need for specialized equipment or invasive procedures makes this test a major advance for peripheral health care facilities. Although the rk39 dipstick test has an ideal format for use in the field, as it is a rapid and simple test not requiring extensive training of the operator, its lower sensitivity limits its use for VL diagnosis among HIV-infected patients. Unsatisfactory results have also been observed in Africa in studies assessing populations with unknown HIV infection status [71], [72], and one possible explanation would be underdiagnosed HIV coinfection.

Molecular diagnoses involving PCR combine several advantages: PCR is minimally invasive, has a high sensitivity and specificity, and is capable of identifying relapses and reinfections in treated VL patients. This is beneficial because the use of a blood test means that invasive diagnostic procedures can be avoided. However, in endemic regions, asymptomatic carriers of *Leishmania* may limit the specificity of the test and its use. In a cross-sectional American study [70], a 6% qPCR positivity (4 out 67 patients) in the control group was observed, which indicates the need for further studies using molecular technique in areas with a high transmission rate of *Leishmania*.

Demonstration of *Leishmania* parasites in bone marrow aspirate or in other biologic specimens, either by visualization or culture, is the most reliable diagnostic technique in the setting of an HIV coinfection. The biological specimen most often used for further parasitological *Leishmania* infection confirmation in Americas is bone marrow aspirate, which, according to available data, exhibits a high sensitivity [13], [70]. Cultures of bone marrow aspirate specimens represent an increase in sensitivity of approximately 7% compared to direct examination, according to a local study [70].

## Treatment and Prophylactic Strategies

Compared to non-HIV-infected patients, an HIV positive status was identified as a risk factor for incomplete clinical response following anti-*Leishmania* therapy in two American studies [63], [73]. To date, no studies in the Americas comparatively evaluated the efficacy of various therapeutic options for VL among HIV-infected patients.

Recently, the policy of the Brazilian Ministry of Health has been modified to include HIV-coinfected patients for treatment with liposomal amphotericin B [11]. However, according to the Brazilian VL guidelines, a total dose of 20 mg/kg, which is currently indicated for the treatment of patients without HIV infection, has also been used for HIV-coinfected patients because of the lack of data about the optimal dose required for these coinfected patients in the Americas. The impact of that change in the Brazilian treatment policy, in addition to the findings of the study using a dose of 40 mg of liposomal amphotericin B, should be evaluated in the near future.

In the Americas, the assessment of a therapeutic response after VL treatment is routinely performed by evaluating the disappearance of fever, the reduction of hepatosplenomegaly, and the normalization of hematological disorders. According to published data, VL relapses in the Americas varied between 10% and 56.5% among series [13], [14], [41]. In agreement with this observation, data from a prospective observational study in the process of being published [63] confirm the high rate of VL relapse; despite secondary prophylaxis with amphotericin B every 15 days, as indicated for patients with CD4 counts less than 350 cells/mm^3^, the observed relapse rate was 37%. Follow-up after VL treatment using PCR is currently being studied in this ongoing Brazilian cohort.

The mortality among the 317 American VL cases published up to December 2013 was 18%, ranging from 8.7% to 23.5% among the published series [14], [41], [73], [74]. Based on a single study that explored conditions associated with death in VL-HIV-coinfected patients [13], acute kidney failure, respiratory distress, hemorrhagic phenomena, and a systemic inflammatory response syndrome were conditions identified as related to fatal outcome.

Similar to what has been observed in other opportunistic infections, antiretroviral therapy (ART) should be initiated as soon as anti-*Leishmania* drugs are tolerated, usually within the second week after VL treatment initiation. Although there is still no clear definition of superiority, a drug combination including a protease inhibitor seems an advantageous option because some such drugs present anti-*Leishmania* action in vitro [75].

If, on the one hand, the widespread use of ART has resulted in dramatic reductions in the incidence of VL-HIV coinfection in southern Europe, it appears to have no protective effect against relapses [42]. Repeated relapses tend to become progressively less acute, more atypical, and less responsive to treatment. Even while on highly active ART, 1-year relapse rates of approximately 30% to 60% have been reported around the world [42].

Due to the lack of local data and based primarily on information about *L. infantum* from European studies, secondary prophylaxis is currently given in Brazil. Amphotericin B every 2 weeks has been the regimen most commonly used. A systematic review performed by [42] also identified the predictors of VL relapse in HIV-infected patients: the absence of an increase in CD4+ T cells at follow-up, a lack of secondary prophylaxis, previous history of VL relapses, and CD4+ T cell counts below 100 cells/µl at the time of primary VL diagnosis. Therefore, it is important to detect and promptly treat concurrent opportunistic infections (especially tuberculosis) and to restore the immune function of coinfected patients with ART. A long period of close monitoring to promptly detect relapses and to maintain secondary prophylaxis might be desirable. Regarding drug resistance, the evidence is scarce, but a higher proportion of *Leishmania*-Sb^+5^-resistant isolates from HIV-infected patients has been observed in Brazil [76].

## Disease Control Activities

Control of VL in the Americas has proved a difficult task. Current control measures are based on weak evidence. Dog culling, spraying insecticides, treatment of human cases, and environmental management have been practiced by national control programs in various combinations and different degrees of intensity and periodicity. The results of those interventions are modest in the best case scenario, and a reliable record of their application in the field is usually lacking. Certainly, the study of interventions on the factors responsible for the rapid spread of VL among the urban population, including the highly vulnerable HIV-infected people, should be prioritized [77]. This must include an understanding of the epidemiology of vectors and of the urban reservoirs, which in the case of the Americas is the domestic dog. Early diagnosis and treatment is essential for the patient but has limited impact on transmission if the primary animal reservoir or insect vectors are not dealt with [78]. Recently, Costa and Cols [79] reported the results of mathematical modeling, which suggest a more rational approach to reduce the burden of canine VL. This effort is part of a revision of the policy for controlling leishmaniasis in Brazil, which could improve the effectiveness of the public health interventions against VL [79].

Development actions and the control of leishmaniases were a commitment made by Member States at the World Health Assembly (WHA), at which resolution WHA 60.13, 2007 was approved. This reinforced the commitment of American states, especially with respect to resolution CD 49.19 2009—PAHO/WHO. Furthermore, efforts are being made to strengthen the surveillance of leishmaniasis in the Americas. For VL-HIV coinfection, a specific field is available in the regional surveillance system for leishmaniasis of PAHO/WHO, which has caused the insertion of this field into the national surveillance systems. At the present time, integrated work between the national leishmaniasis and AIDS control programs is urgently needed to strengthen the surveillance of *Leishmania*-HIV coinfection to improve the delivery of early interventions that can reduce the lethality of this condition.

## Knowledge Gaps, Operational Challenges, and Research Priorities

This narrative review of evidence related to VL-HIV coinfection in Latin America revealed that much work remains to be performed to clarify the most relevant clinical and epidemiological aspects of this condition. The exact burden of the disease remains largely unknown because most countries lack effective surveillance systems, and even countries such as Brazil with a consolidated surveillance system suffer from significant underreporting. Strong surveillance will certainly contribute to improved data quality for decision makers in this complex scenario.

The impact of the HIV/AIDS pandemic is not only changing the natural history of leishmaniasis but also increasing the risk (100 to 1,000 times) of developing VL in endemic areas [6], reducing the therapeutic response [56], and increasing the possibility of relapse [42]. Where HIV counseling and access to ART are available, all VL patients should be screened for HIV, as currently recommended in Brazil, to improve the treatment options for HIV-coinfected patients.

While the clinical manifestations of VL in HIV-coinfected patients without severe immunosuppression are similar to those in immunocompetent patients, atypical clinical features can be found in patients with a low CD4+ T lymphocyte count. In the latter group, physicians should suspect VL even in the absence of classical signs such as splenomegaly [80]. In fact, in most countries where HIV and *Leishmania* are endemic, VL should be considered a condition related to immunosuppression, which could indicate the need for anti-HIV infection tests and antiretroviral therapy initiation.

The diagnosis of VL in HIV-infected patients may not be straightforward due to the clinical variations from nonspecific to atypical features found in many of the cases. A substantial proportion of VL-HIV patients may present with other opportunistic infections, which complicate the clinical diagnosis. In suspected cases of leishmaniasis in HIV-infected patients, combined diagnostic investigations in parallel are recommended to confirm the diagnosis [81]. In Brazil, serological tests provided by the public healthcare system are a rapid test based on the antibody against the recombinant antigen K-39 and IFAT, both tests with insufficient performance in immunocompetent VL patients and with the lowest sensitivity among HIV-infected patients [70]. It is worrying to not use a specific diagnostic screening for VL among HIV-infected patients. Such screening should include a wider range of diagnostic assays, including the use of more sensitive tests, such as DAT and molecular tests [70]. The benefit of using more invasive methods for VL diagnosis, such as bone marrow puncture, should be underscored in HIV-infected patients.

With a global perspective, it is a major challenge to develop a new drug for the treatment of VL. Clinical research to evaluate new drugs and combinations of drugs to reduce the duration of treatment for all clinical forms of leishmaniasis remains a high priority. Compared to Asia and Africa, therapeutic choices in Latin America are even more limited because miltefosine and paromomycin are not available options. An additional challenge to be tackled is the regional variations in the therapeutic response to drugs, such as miltefosine, due to unknown factors that could be related to *Leishmania* strains or specific pharmacokinetics and pharmacogenomics profiles of the affected population. Combination regimens should be tested in coinfected patients, as they may improve treatment efficacy and reduce toxicity. Currently, there is no successful therapy for VL-HIV-coinfected patients [56]. Moreover, few clinical studies addressing the efficacy of drugs in coinfected patients have been conducted outside of the Mediterranean area even in the face of evidence that the total dose requirements for the treatment of VL vary by region. Current VL-HIV coinfection treatment in Latin America is based on rather weak scientific evidence. There is no consistent information comparing different treatment regimens in the Americas, including different formulations and the optimal dose of amphotericin B. One crucial point would be the understanding of the immunopathological mechanisms, such as the role of bacterial translocation in the immunoactivation in these patients [29], which may lead to antibiotic use in selected cases.

Finally, VL-HIV coinfection research priorities must include well-designed validation studies to evaluate the accuracy and reliability of the available diagnostic tests and their potential combinations as well as well-designed and well-conducted clinical trials for disease treatment and secondary prophylactic schedules. In spite of the increasing incidence of VL-HIV-coinfected patients, the number of cases for conducting powerful trials will still require a multicentric approach. Thus, the networking effort to put together the reference centers where most of the coinfected patients look for treatment is crucial for the success of any trial initiative. Networking could also offer the opportunity for the inception of a cohort of VL-HIV-coinfected individuals to explore hypotheses linked to prognostic issues, such as the role of HIV protease inhibitors in decreasing the incidence of VL relapses.

We strongly support the formation of a Latin American network on coinfection of *Leishmania*-HIV to study and better understand the clinical manifestations and immunopathogenic aspects of VL in immunosuppressed patients as well as to coordinate and validate diagnostic and therapeutic trials. Such trials would result in more specific and effective public policies on diagnosis and treatment, reducing the VL-HIV coinfection fatality rate observed in this region.

**Box 1. Key Learning Points**

1. There is an increase in visceral/leishmaniasis coinfection in Latin America due to overlapping areas of both diseases, and the profile of coinfected patients is male and adults aged between 20 to 49 years. We suggest HIV testing be included as part of the routine evaluation of visceral leishmaniasis patients in Latin America, mainly in young adults.
2. The impact on the immune system caused by *Leishmania* and HIV infections contributes to progression of the diseases. The chronic activation caused by *Leishmania* infection can constitute an additional factor for worsening the clinical condition of HIV patients, having a consequent enhanced viral load, T cell proliferation, and cell death induced by activation. The HIV infection also impairs the *Leishmania*-specific T lymphocyte proliferation and IFN-γ production, favoring the spread of the *Leishmania* parasite.
3. Clinical presentation of visceral leishmaniasis in HIV-infected patients is similar to patients without HIV infection; however, diarrhea appears to be common in coinfected patients, and hepatomegaly appears to be less common in coinfected patients. Cutaneous lesion caused by *L.(L.) infantum*, characterized as PKDL-like, is rare in HIV-infected patients from Latin America.
4. Regarding diagnostics of visceral leishmaniasis in HIV-infected patients, parasitological tests are more sensitive, but serological tests have variable sensitivity according to the antigen and method used. Based on data from Latin America, we suggest using parasitological tests to confirm diagnosis of visceral leishmaniasis in HIV-infected patients.
5. Visceral leishmaniasis in HIV-infected patients presents more relapses and lethality. The predictors of VL relapses are absence of an increase in CD4+ T cell counts at follow-up of patients, a lack of prophylaxis, a previous history of visceral leishmaniasis, and CD4+T cell counts below 100 cells/mL at the time of primary VL diagnosis. Antiretroviral therapy, including protease inhibitor, should be initiated as soon as possible.

**Box 2. Top Five Papers**

1. Maia-Elkhoury AN, Alves WA, Sousa-Gomes ML, Sena JM, Luna EA (2008) Visceral leishmaniasis in Brazil: Trends and challenges. Cad Saude Publica 24: 2941–2947.
2. Santos-Oliveira JR, Regis EG, Giacoia-Gripp CB, Valverde JG, Alexandrino-de-Oliveira P, et al. (2013) Microbial translocation induces an intense proinflammatory response in patients with visceral leishmaniasis and HIV type 1 coinfection. J Infect Dis 208: 57–66.
3. Cota GF, de Sousa MR, de Freitas Nogueira BM, Gomes LI, Oliveira E, et al. (2013) Comparison of parasitological, serological, and molecular tests for visceral leishmaniasis in HIV-infected patients: A cross-sectional delayed-type study. Am J Trop Med Hyg 89: 570–577.
4. Cota GF, de Sousa MR, Rabello A (2011) Predictors of visceral leishmaniasis relapse in HIV-infected patients: A systematic review. PLoS Negl Trop Dis 5: e1153.
5. Cota GF, de Sousa MR, Fereguetti TO, Rabello A (2013) Efficacy of anti-leishmania therapy in visceral leishmaniasis among HIV infected patients: A systematic review with indirect comparison. PLoS Negl Trop Dis 7: e2195.
